# “Knowledge, recommendation, and beliefs of e-cigarettes among physicians involved in tobacco cessation: A qualitative study”

**DOI:** 10.1016/j.pmedr.2017.07.012

**Published:** 2017-08-05

**Authors:** Binu Singh, Mary Hrywna, Olivia A. Wackowski, Cristine D. Delnevo, M. Jane Lewis, Michael B. Steinberg

**Affiliations:** a Department of Health Education & Behavioral Science, Center for Tobacco Studies, Rutgers School of Public Health, 683 Hoes Lane West Piscataway, NJ 08854, United States; b Department of Medicine, Robert Wood Johnson Medical School, Rutgers University, 125 Paterson Street, New Brunswick, NJ 08901, United States

**Keywords:** E-cigarette, Harm reduction, Smoking cessation, Physicians, Qualitative

## Abstract

Physicians are rated the most trustworthy source of information for smokers and thus play an increasing role in disseminating information on e-cigarettes to patients. Therefore, it is important to understand what is currently being communicated about e-cigarettes between physicians and patients. This study explored the knowledge, beliefs, communication, and recommendation of e-cigarettes among physicians of various specialties. Semi-structured interviews were conducted in early 2016 with 35 physicians across five different specialties. Interviews were transcribed and coded for the following deductive themes: (1) tobacco cessation recommendation practices, (2) knowledge of e-cigarettes, (3) communication of e-cigarettes with patients, (4) recommendation of e-cigarettes, and (5) general beliefs about e-cigarettes. Physicians across all specialties reported having conversations with patients about e-cigarettes. Conversations were generally prompted by the patient inquiring about e-cigarettes as a cessation method. Overall, physicians felt there was a lack of information on the efficacy and long term health effects but despite lack of evidence, generally did not discourage patients from trying e-cigarettes as a cessation device. Although physicians did not currently recommend e-cigarettes over traditional cessation methods, they were open to recommending e-cigarettes in the future if adequate data became available suggesting effectiveness. Patients are inquiring about e-cigarettes with physicians across various specialties. Future research should continue to study physicians' perceptions/practices given their potential to impact patient behavior and the possibility that such perceptions may change over time in response to the evidence-base on e-cigarettes.

## Highlights

• Physicians across specialties are having conversations with patients about ecigs.
• Generally did not discourage patients from trying ecigs as a cessation device.
• Physicians open to recommending ecigs for cessation in the future.
• Adolescent use major cause of concern for physicians.

## 1 Background

Electronic cigarettes have garnered much attention among the public in recent years due to rising sales and contentious harm-reduction debates. Data suggests that the majority of e-cigarette users are current and former cigarette smokers and use among adult smokers is increasing (Giovenco et al., 2014, Wilson and Yang, 2016). Many smokers perceive these products to be less risky than cigarettes and some use them as an alternative to cigarettes (Tan et al., 2014, Goniewicz et al., 2013, Etter and Bullen, 2014, Wackowski and Delnevo, 2016). However, many smokers also believe that e-cigarettes are not harmless and are interested in safety information (Wackowski et al., 2015). While research on health effects of e-cigarettes is still in its infancy, one recent study found that long term e-cigarette only use was associated with lower levels of carcinogens and toxins when compared to cigarette only use; however, nicotine intake was roughly similar between the two products (Shahab et al., 2017). Despite limited evidence on safety and efficacy, many smokers have turned to e-cigarettes for quitting smoking, to use in areas where cigarettes are prohibited, and as a healthier alternative to cigarette smoking (Soule et al., 2016, Saddleson et al., 2016, Wackowski et al., 2016) with some finding e-cigarettes more satisfying and helpful in quitting than FDA-approved cessation medications (Steinberg et al., 2014, Rahman et al., 2015, Harrell et al., 2015).

As e-cigarette use proliferates, smokers may increasingly turn to their physicians with questions regarding these products. Physicians have a unique role in smoking cessation as they treat smoking patients on a regular basis over years, amassing medical histories, and establishing provider-patient relationships. Furthermore, physician advice has been recognized as a major determinant in making an attempt to quit (Fiore et al., 2000). In the context of health information, research suggests that while individuals reported receiving significant amounts of information from television, internet, and elsewhere, the most widely accessed and trusted source was their physician (Smith, 2011). Similarly, physicians may be an increasingly important source to balance the widely available information regarding e-cigarettes from industry advertising, media reports, and celebrity endorsements, which may not be evidence-based or scientifically accurate. A previous study of smokers found that most would turn to physicians for e-cigarette safety information and rated physicians as the most trustworthy source of such information (Wackowski, 2014).

Although the literature on this topic is limited, initial findings suggest that patients are talking with their physicians about e-cigarettes. Previous studies have found that between 7 and 27% of adult smokers have talked to their physicians about e-cigarettes (Wackowski et al., 2015, Berg et al., 2015, Kollath-Cattano et al., 2016). In a national web-based survey of 158 physicians, nearly two-thirds (65%) of physicians reported being asked about e-cigarettes by their patients and almost a third (30%) reported that they recommended e-cigarettes as a smoking cessation tool (Steinberg et al., 2015). Moreover, patient inquiries about e-cigarettes significantly increased over the course of the study.

The aim of the current study was to explore physicians' knowledge, perceptions, and communications regarding e-cigarettes via semi-structured interviews with physicians from a variety of specialties. To our knowledge, this is the first qualitative study to include physicians of various specialties who may be directly involved in smoking cessation and treating smoking-related conditions.

## 2 Methods

### 2.1 Subject recruitment and interview process

Using the market research company GfK, 35 physicians were recruited to participate in semi-structured interviews through the Physicians Consulting Network (PCN). The PCN is a database which includes over 70,000 physicians who have opted in to be contacted with research opportunities. All physicians in the PCN are verified via medical education numbers through the American Medical Association. GfK contacted, screened, and scheduled interviews with all physicians participating in the study. More information on GfK can be found at their website (http://www.gfk.com/about-gfk/about-gfk/, n.d.). Certain specialties (i.e., Primary care, Obstetrics/Gynecology (OB/GYN), Pulmonology, Cardiology, and Oncology) were targeted for inclusion based on their regular interaction with smokers as well as their role in tobacco cessation and treating tobacco-related diseases. We included primary care physicians because they are the most common physician specialty and thus a likely group to encounter e-cigarette questions. We also included cardiologists, pulmonologists, oncologists and OB/GYNs because smoking is a particularly important risk factor in the patients seen by these specialists and as such they may have unique attitudes toward e-cigarettes and the issue of tobacco harms reduction. Other eligibility requirements included: (1) provided direct patient care, (2) saw smokers in their clinical practice, and (3) ever heard of e-cigarettes. We set a target sample size of 35 based on previous interview studies conducted by the research team (Wackowski et al., 2016) and published on this topic (El-Shahawy et al., 2016). By the 35th interview, no new major themes or unique responses were emerging (i.e., reaching saturation) and thus we did not expand the sample size. Participants received a $250–350 gift card for participating in the 20–40 min telephone survey, depending on specialty.

Interviews were semi-structured in nature and followed a guide developed by the research team. Questions were based on review of relevant tobacco control literature, trade sources, and the investigators' knowledge of the e-cigarette industry. The interview guide included questions covering the target themes of (1) tobacco cessation recommendation practices, (2) knowledge of e-cigarettes, (3) communication of e-cigarettes with patients, (4) recommendation of e-cigarettes, and (5) general beliefs about e-cigarettes. All interviews were audio recorded and transcribed. The interviews were conducted between January and February of 2016 by multiple members of the research team (BS, MBS, MH, and MJL).

### 2.2 Coding and analysis

Coding was informed using the “framework analysis” method in which themes are developed both from the research questions/interview guide as well as through the responses of research participants (Rabiee, 2004). Two members of the research team (B.S., M.S.) read through the interview transcripts and developed codes based on deductive themes linked to the study's aims, interview guide questions, and inductive themes arising from repeated transcript readings. Transcripts were coded by identifying mentions of major themes as well as highlighting representative quotes. Transcripts were primarily coded by one member of the research team (B.S.) using Atlas.ti qualitative software. A sample of transcripts was reviewed by another team member (M.H.) for agreement in assignment and discrepancies were identified, discussed, and resolved. Coding and analysis was conducted between May–October 2016.

## 3 Results

### 3.1 Physician demographics

A total of 35 physicians were interviewed including 10 primary care physicians, 10 OB/GYN, 5 cardiologists, 5 pulmonologists, and 5 oncologists. The mean age of participants was 55.5 years (range, 44–66) and mean years of practice was 25 years (range, 11–36). Additional demographic information is presented in Table 1.

**Table 1:** Demographics.

Variable	Participants
Gender	
Male	27 (77%)
Female	8 (23%)
Race	
White	33 (94%)
African-American	1 (3%)
Asian	1 (3%)
Geographic region	
West	8 (22.9%)
Midwest	10 (28.6%)
Northeast	8 (22.9%)
South	9 (25.6%)

### 3.2 e-Cigarette knowledge/awareness

Physicians across all specialties reported some basic knowledge of e-cigarettes. Aspects of e-cigarettes commonly reported were: e-cigarettes come in various flavors, contain known and unknown chemicals, contain nicotine, and are federally unregulated (which was still the case during these interviews). In regards to flavors, physicians reported that their likely purpose was to make vaping a “…pleasant experience” (Participant 2, Primary Care) and as a way to appeal to a younger population.

“What's troublesome about that [flavors] is my impression is it's got to be aimed at kids. Kids…they're into flavors.”

(Participant 13, Oncologist)

When asked about the demographics of e-cigarette users, the majority of physicians identified adolescents and young adults as the primary users of e-cigarettes.

“My other sense I get is that it probably became somewhat of a fad…adolescents and younger adults thought this was a cool thing to do instead of smoking cigarettes.”

(Participant 4, OB/GYN)

“I would say 20–30 year old people…I'm not seeing 40, 50, 60 year olds using e-cigarettes.”

(Participant 32, Primary Care)

Physicians were aware that e-cigarettes contained nicotine and other chemicals but responses were mixed when asked about studies they had seen on safety and efficacy. While some physicians reported not having seen any studies (“Articles on e-cigarettes appear infrequently in the different medical journals that I peruse,” Participant 6, Oncologist), nine physicians, including all five pulmonologists, did speak about studies they had seen in medical journals (e.g., Journal of the American Medical Association, American Journal of Respiratory and Clinical Care).

“In regards to the data on the effectiveness of e-cigarettes I guess there's two placebo control studies out there that show that it helps and there's one that compares it to nicotine patches and it's a wash. I think it was better for the first month but when you looked at three and six it was about the same.”

(Participant 8, Pulmonologist)

In addition to scientific studies, a pulmonologist also noted hearing about various e-cigarette topics in the popular media, including “…articles talking about their success rates helping people to quit, the dangers of e-cigarettes, what they contain, whether they lead people to more smoking rather than less smoking. There are all kinds of articles out there.” (Participant 3, Pulmonologist)

### 3.3 Patient communication of e-cigarettes

Of the 35 physicians interviewed, only 2 (both OB/GYNs) reported *never* having discussed e-cigarettes with patients. Among the physicians who reported conversations with patients about e-cigarettes, the conversations were mainly prompted by the patient informing the physician that he/she had tried or wanted to try e-cigarettes to quit smoking and he/she sought physician advice on e-cigarette safety and efficacy as a cessation device. In the few instances where a physician initiated the conversation, the exchange was the result of seeing a patient physically holding an e-cigarette or identifying themselves as a user of e-cigarettes.

“I think patients bring it up because they want my approval or my okay that it's safe and better for them. I think that's really the truth. I think they're looking for some affirmation or assurance it's better for them than smoking.”

(Participant 30, Cardiologist)

When asked by patients about the safety and efficacy of e-cigarettes, physicians from all specialties were open with patients about the lack of information on the long-term health effects of e-cigarettes and that they cannot definitively say whether it is a good or bad product for smoking cessation based on the evidence available.

“I tell them the jury is still out. We don't know about the long term safety, we don't know about the efficacy.”

(Participant 2, Primacy Care)

### 3.4 Physician recommendations

While physicians informed patients that there is a lack of data on the safety and efficacy of e-cigarettes and did not actively recommend them, they did not discourage patients from using them. This was particularly true for patients who had not succeeded with commonly recommended cessation products (i.e., Chantix, nicotine replacement, cessation programs, etc.) and expressed interest in trying e-cigarettes or were currently using e-cigarettes for cessation.

“No, I'm not recommending it. I'm saying it's an option for people who have already tried it and people who are thinking about trying it. I'm not looking to promote e-cigarettes but I would not disapprove of it especially if they have tried other products and have not succeeded.”

(Participant 3, Pulmonologist)

“Yeah but suppose they said to me ‘I've already tried that. I've tried the patch, I've tried the gum and it hasn't worked.’ So in that situation I would definitely say okay if you've tried that and that hasn't worked maybe it might be worthwhile to give e-cigarettes a shot to see whether you're any more successful.”

(Participant 4, OB/GYN)

When asked about recommending e-cigarettes in the future, physicians of all specialties said they would consider recommending e-cigarettes for cessation in the future if adequate data such as randomized trials comparing the efficacy of e-cigarettes with other cessation products showed e-cigarettes were equally or more effective than currently recommended products. However, five physicians (one of each specialty interviewed) stated that they would not recommend e-cigarettes in the future *even if* data became available. One oncologist said:

“Based on what I′ve heard and read about them, I don't think so. It seems like they're actually, like I said, kind of dangerous.”

(Participant 23, Oncologist)

Approximately half of physicians believed e-cigarettes could be an effective cessation aid, including all cardiologists interviewed and over half of primary care physicians. Physicians' beliefs were based on one or more studies they had seen in which e-cigarette users had a favorable smoking quit rate as well as anecdotal evidence of having seen their patients or family/friends quit or reduce the amount of cigarette smoking with the use of e-cigarettes.

“I think there was a Canadian study that showed and the number that comes to mind was about a 20% quit rate on e-cigarettes compared to placebo.”

(Participant 2, Primary Care)

“I've had some patients who have switched to e-cigarettes and they decreased the smoking amount over time to quit smoking”

(Participant 21, Primary Care)

The other half of physicians either did not believe in the efficacy of e-cigarettes for smoking cessation or were undecided. Among the most common reasons for believing e-cigarettes were not effective for cessation was the behavioral aspect of the hand to mouth motion, expressed by eleven specialist physicians. They believed that mimicking the motion of smoking cigarettes could be a deterrent to cessation.

“And then there's the psychological process of holding the device and inhaling. To me, inherently, that would not be as good a product to get somebody off cigarettes”

(Participant 11, Cardiologist)

### 3.5 General beliefs about e-cigarettes

Physicians were asked about their general beliefs on a variety of topics surrounding e-cigarettes such as their relative harm compared to cigarettes and nicotine replacement therapies (NRT) and emerging public health concerns of e-cigarettes. The majority of physicians felt that e-cigarettes were less harmful than traditional cigarettes but more harmful than nicotine replacement therapies, such the nicotine patch and gum.

“I think the patch and the gum are safer, there's no inhaling of anything and in the patch there are no other chemicals that we need to be worried about.”

(Participant 27)

Despite physicians responding that the relative harm of e-cigarettes was less than that of traditional cigarettes, there were still safety concerns surrounding the use of e-cigarettes expressed by physicians from the various specialties. For example, pulmonologists were particularly concerned about the effects of inhalation of the e-cigarette liquid on the lungs while OB/GYNs were concerned about the effect of the nicotine on pregnant patients.

The major public health concern mentioned was the appeal and use of e-cigarettes by adolescents, mentioned over 40 times across the 35 interviews. Physicians' concerns included increased e-cigarette use by adolescents, potential gateway effects, and targeted e-cigarette marketing to young populations.

“I am most concerned about gateway to other tobacco products and also impact on minors. I think that's a big one and I'm very, very concerned about that.”

(Participant 2, Primary Care)

“That is a bit of a concern because like I said sometimes I have teenagers come in and tell me they use them, they call it vaping…so that does somewhat bother me that yes we are sort of creating a culture of another habit.”

(Participant 33, Primary Care)

In addition, physicians were also concerned with the contents of the vapor emitted from e-cigarettes and its potential harm to others.

“The other concern I have is about the effects of the vapors on by-standers. We are [aware] of the effects of second-hand tobacco smoke on individuals and innocent by-standers. We don't know what the effect of [e-cigarette vapor] would be… they look like a chimney coming out… I don't want to walk past that…I don't know what the effect of it is, so I'm concerned about that potentially affecting me, affecting you, and affecting others who don't want to smoke.”

(Participant 22, OB/GYN)

When asked generally whether they had an overall positive, neutral, or negative view of e-cigarettes, only two physicians (an oncologist and primary care physician) stated they had an overall positive view based on the potential of e-cigarettes as a cessation aid, 17 physicians had an overall negative view, and eight had an overall neutral view. In addition, 15 physicians stated that their views on e-cigarettes changed over time, with most of these (11 physicians) reporting their view become more negative over time, and only 4 reporting that their view became more positive over time.

“I thought they were an interesting phenomenon originally…I don't really follow the research…just what I come across in the lay literature. There are increasing concerns about what is in that vapor other than just nicotine so I am more concerned now than I was originally…there is more and more rumblings about the potential negative effects of the product. It's just not steam and nicotine, there's more to it.”

(Participant 15, OB/GYN)

“Initially when I heard of e-cigarettes; I thought it was just like a low nicotine cigarette. Once I got knowledge about what it is, the ingredients, what's created when the liquid is heated up, then it changed I said hey this is not a cigarette. This is something that has nicotine but not the other stuff that tobacco has. So yeah on that end it's been more of a positive.”

(Participant 19, OB/GYN)

## 4 Discussion

This qualitative pilot study of physicians' beliefs and communication with patients regarding e-cigarettes demonstrated that conversations with patients regarding e-cigarettes are occurring frequently with physicians of various specialties. Most believed e-cigarettes to be less harmful than cigarettes, which is an important factor if physicians are to play a role in their use for tobacco harm reduction. However, physicians across groups also expressed concerns about their safety and efficacy. The focus of safety concerns about e-cigarettes were somewhat different by physician specialty, with pulmonologists expressing concerns over vapor inhalation while OB/GYNs expressed concerns about nicotine content and e-cigarette use during pregnancy. In addition, only about half of physicians believed e-cigarettes could be an effective cessation aid. Physicians indicated speaking with patients about these concerns and these reservations appear to currently limit their active recommendation of e-cigarette use to their patients.

On the other hand, it was found that while physicians did not actively recommend e-cigarettes to patients, they also did not discourage interested patients from trying e-cigarettes as a cessation device, particularly among those who failed to quit with other smoking cessation methods. This was consistent with another study that found primary care providers were more inclined to recommend e-cigarettes to patients with failed quit attempts (El-Shahawy et al., 2016). Physicians were also open to patients who were smokers with existing chronic conditions (i.e. lung cancer, heart disease, chronic obstructive pulmonary disease) to switch to e-cigarettes as a means of harm reduction.

Consistent with results from previous qualitative studies with physicians (El-Shahawy et al., 2016, Gorzkowski et al., 2016), physicians in this study, across disciplines, reported wanting to see more scientific studies on e-cigarette safety and efficacy to better inform patients and make decisions on recommendations. Some specifically cited a need for data from randomized clinical trials (RCTs) comparing the efficacy of e-cigarettes with other cessation products. While such studies may indeed be informative, building a RCT research base will take time and their generalizability may be limited given the wide range of vaping products available on the market relative to traditional NRT products. Furthermore, such trials may underestimate the impact of e-cigarette harm reduction effectiveness in the “real world”, as consumer specific subjective preferences, such as the ability to customize flavor type and nicotine level, appear to be important factors in e-cigarettes' appeal. As such, physicians should also be informed of observational research about the harm reduction impact of e-cigarettes, and about their market and consumer appeal to smokers relative to traditional smoking cessation treatments, factors which are also important in their actual use and real world impact on smoking cessation (Steinberg et al., 2014, Steinberg et al., 2016).

Although efforts were made to recruit a geographically diverse sample that was also representative of specialties involved in tobacco cessation and harm reduction, physicians' knowledge, attitudes, and beliefs about e-cigarettes may also be influenced by their medical training; workplace, community, and state tobacco control policies; and regional tobacco and e-cigarette industry practices. Limitations of this study included the small sample size and limited demographic diversity (largely white males). Given that authors were not directly involved in recruiting participants, the number and demographics of physicians who declined to participate is also unknown.

## 5 Conclusion

Overall, patients are contacting physicians of various specialties for information on e-cigarettes. Despite the lack of studies on the cessation efficacy and long-term effects of e-cigarettes, physicians are generally not discouraging patients from trying e-cigarettes as a cessation method or form of harm reduction. Future research should continue to study physicians' e-cigarette related perceptions and practices given the potential for physicians to influence patient behavior and for physicians' advice to change over time in response to the evolving scientific evidence on e-cigarettes. In addition to individual physicians, it is important to understand how leading medical organization positions' may influence physicians' perceptions of e-cigarettes. For example the American Lung Association and American College of Physicians have both voiced concerns about the safety and efficacy of e-cigarettes and encouraged physicians to continue using FDA-approved cessation techniques, whereas organizations such as the American Society of Clinical Oncology, while also stating concerns regarding e-cigarettes and regulation, have not taken a definitive stance on recommending e-cigarettes for smoking cessation.

## List of abbreviations

PCN = Physicians Consulting Network.

## Appendix A PACE physician interview guide

Inclusion criteria for recruitment:

1. Physicians from the 3 specialty areas (Primary care, Tobacco caused diseases (pulm, cardio, onc), OB/GYN who see pregnant women).
2. Physicians involved in direct patient care.
3. Physicians who see tobacco users in their patient care.
4. Physicians who have heard of e-cigarettes.

Guide:

1. What is your medical specialty?
2. Do you see smokers in your clinical practice? – If NO – end interview.
3. What do you see as your role in working with smokers?
  a. PROBE – if not mentioned, “What about Helping them quit?”
4. What kinds of things do you recommend to help people trying to quit?

Now let's talk about e-cigarettes.

5. Have you ever heard about e-cigarettes?
  a. What have you heard about e-cigarettes?
    i. PROBE:
      1. What have you heard about the vapor from e-cigarettes? Flavors? Nicotine?
      2. Do you have a sense as to who uses e-cigarettes?

Now, let's talk about conversations you might have had with patients.

6. What kinds of discussions have you had with patients about e-cigarettes? Who starts the conversation?
7. Why do they generally bring it up?
  a. PROBE: Is it because they want to quit smoking?
8. Can you give examples of what you say when asked about e-cigarettes?
9. Do you ever recommend that a patient use e-cigarettes?
  b. IF YES
    i. What is your reason for recommending them?
    ii. What do you tell patients about e-cigarettes?
  c. If NO
    i. Are you likely to do this in the future?
    ii. What additional information would you need to start recommending them?
    iii. PROBE: What if studies showed that they were safer than regular cigarettes?
10. Why do some patients use e-cigarettes?
  a. PROBE – To stop smoking? To get through a time where they temporarily can't smoke?

We are going to change gears a bit now and talk about beliefs that people have about e-cigarettes.

11. What do you think about the relative harm of e-cigarettes compared to smoking regular cigarettes?
  a. How is their harm compared to nicotine replacement (for example, nicotine gum)?
12. Some people say e-cigarettes can help people stop smoking. What do you think?
13. People have mentioned other concerns about e-cigarettes apart from individual health impacts. These include renormalizing smoking behavior in society, appealing to young people, or as a gateway to other tobacco products. What do you think?
14. Have you ever tried an e-cigarette?
  d. If YES - What was your experience?
